# Barriers to and Facilitators of Implementing Overnight Nursing Teleconsultation in Small, Rural Long-Term Care Facilities: Qualitative Interview Study

**DOI:** 10.2196/71950

**Published:** 2025-05-07

**Authors:** Veronique Nabelsi, Véronique Plouffe, Marie Chantal Leclerc

**Affiliations:** 1 Département des sciences administratives Université du Québec Québec Canada; 2 Département des sciences comptables Université du Québec en Outaouais Gatineau Canada; 3 Département des sciences infirmières Université du Québec en Outaouais Gatineau Canada

**Keywords:** teleconsultation, long-term facilities, nursing, barriers and facilitators, rural, telehealth, qualitative, pilot study, Quebec

## Abstract

**Background:**

Teleconsultation has expanded rapidly in recent years, especially during the COVID-19 pandemic, and has become standard practice among physicians. The benefits of teleconsultation, namely, improving access to care, ensuring continuity and quality of care, increasing patient satisfaction, and reducing costs and wait times, are well documented. However, its use in nursing practice, especially in long-term care settings, remains underresearched despite its significant transformative potential, particularly in resource-limited and rural settings, where it could address major challenges such as nursing shortages and access to care.

**Objective:**

This study aimed to identify barriers to and facilitators of implementing overnight nursing teleconsultation in rural residential and long-term care centers in Quebec, Canada (*centres d’hébergement et de soins de longue durée* [CHSLDs]), with ≤50 beds.

**Methods:**

A 6-month pilot project was rolled out sequentially in 3 rural CHSLDs in 2 administrative regions of Quebec between July 2022 and March 2023. A total of 38 semistructured interviews were conducted with managers (n=27, 71%), nursing staff members (n=9, 24%), and resident committee presidents (n=2, 5%) between February 2023 and July 2023.

**Results:**

The study identified several barriers to the implementation of teleconsultation. The main barriers reported included union opposition (managers: 23/27, 85%), network instability (resident committee presidents: 2/2, 100%), limited technology skills (nursing staff members: 7/9, 78%), a perceived increase in workload (nursing staff members: 8/9, 89%; resident committee presidents: 2/2, 100%), and a low volume of teleconsultations (nursing staff members: 8/9, 89%). Despite the barriers, participants also identified key facilitators. These included the care setting (nursing staff members: 9/9, 100%; managers: 21/27, 78%), buy-in from senior management and managers (managers: 27/27, 100%; resident committee presidents: 2/2, 100%), collaboration between the departments (nursing staff members: 9/9, 100%), nursing staff motivation (nursing staff members: 9/9, 100%), and improvements in professional practices (nursing staff members: 8/9, 89%). Finally, the relative benefits of teleconsultation, such as enhanced mutual vision, faster assessment of clinical situations, improved resident care management quality, and greater flexibility and safety, were unanimously recognized (38/38, 100%) as contributing to its acceptability and potential for success.

**Conclusions:**

This study provides an in-depth understanding of the barriers to and facilitators of implementing overnight nursing teleconsultation in small rural CHSLDs. This constitutes a sound basis for developing tailored strategies aimed at overcoming identified barriers and optimizing facilitators. The results also provide practical guidelines for decision makers, highlighting the need to adapt implementation approaches to the unique context of each facility. Furthermore, this study highlights the importance of further research to broaden our knowledge on the dissemination and scale-up of health care innovations. This includes the development of learning health systems capable of responding in an agile and effective way to the needs of rural and vulnerable populations both in Quebec and elsewhere.

## Introduction

### Background

Teleconsultation has evolved considerably in recent years, especially during the COVID-19 pandemic, when it became standard practice for general practitioners and specialists alike [1-3]. Numerous studies have demonstrated its effectiveness in improving access to care [4-6], ensuring continuity [7,8] and quality of care [9,10], increasing patient satisfaction [11-15], and reducing costs [16-18] and wait times [2,19-22]. Moreover, internet-based clinical support initiatives between novice and expert professionals are being implemented in both urban [23-25] and rural [26,27] settings specifically to address challenges related to the shortage of qualified health care workers.

Despite the increasing adoption of teleconsultation, its use in nursing practice remains largely unexplored [28-31], even though its potential to transform care, including in long-term care centers, is well recognized [32,33]. This potential is even more significant in rural and resource-limited settings, where access to health care services remains a major challenge [34-37].

The lack of data on the use of teleconsultation in nursing practice is concerning as nurses play a key role in the continuum of care, especially in rural settings and long-term care facilities, where they are often patients’ first point of contact [38-40]. Although recent studies have focused on the facilitators of and barriers to implementing geriatric teleconsultation in home nursing care [33,41], its impact on nurses’ and nursing assistants’ workflows [35], and the costs and cost savings associated with its use in residential and long-term care centers [34], these studies remain limited.

To date, studies have not yet provided a comprehensive picture of the effectiveness and impact of teleconsultation in nursing practice. Thus, in-depth exploration of this approach is essential to optimizing its benefits and supporting nursing professionals in the adoption and integration of this technological innovation.

### Context

This research gap is of concern in residential and long-term care centers (*centres d’hébergement et de soins de longue durée* [CHSLDs]) in Quebec, Canada, where continuity and quality of care are essential to residents’ well-being. Directives from the Ministry of Health and Social Services (Ministère de la Santé et des Services sociaux [MSSS]) and guidelines from the Order of Nurses of Quebec (Ordre des infirmières et infirmiers du Québec) stress the need to ensure the presence of nurses 24 hours a day in these facilities. However, smaller CHSLDs, namely, those with ≤50 beds, face major challenges in meeting this requirement due to nursing staff shortages, especially during night shifts. These frequent periods when no resources are available expose residents to increased risks to their safety and well-being [42,43]. This situation is even more critical in semiremote and remote areas, where difficulties associated with recruiting and retaining nursing staff exacerbate the challenges related to access and quality of care. A better understanding of the opportunities offered by teleconsultation could help alleviate these structural challenges and help build the CHSLDs’ capacity in these settings.

As a response to these challenges, the National Directorate of Nursing Care and Services of the MSSS has initiated a pilot project to assess the impact of access to overnight nursing teleconsultation in rural CHSLDs with ≤50 beds. This initiative is based on the implementation of nursing teleconsultation, a promising solution to reinforce continuity of care and reduce regional disparities in access to health care services in Quebec.

Recent studies have shown that teleconsultation can mitigate limitations related to the lack of on-site nursing staff and offer real-time clinical support, thus reducing the risk of adverse events during periods of understaffing [35,44,45].

In line with this approach, this project explored innovative solutions to build resilience in long-term care systems and respond more effectively to the growing challenges associated with nursing shortages.

### Objectives

The aim of this study was to identify barriers to and facilitators of implementing overnight nursing teleconsultation in rural Quebec CHSLDs with ≤50 beds. Specifically, this study aimed to gather the views of managers, nursing staff, and resident committee presidents. Exploring these viewpoints will fill a significant gap in the current literature and suggest critical avenues to support the successful integration of teleconsultation in long-term care settings.

## Methods

### Study Design and Setting

The 6-month pilot project was rolled out in 3 rural CHSLDs located in 2 administrative regions of Quebec. The regions were selected by the MSSS for their alignment with the project’s outlined criteria, which include facilities located in semiremote and remote areas, those already experiencing nursing shortages during the night shift, and those reporting issues and risks related to these shortages. In addition, at least 30% of all CHSLD facilities in the territory have a capacity of ≤50 beds. The rollout was conducted sequentially from July 2022 to March 2023 at different sites. Given the innovative nature of the pilot project, an exploratory qualitative study was conducted to identify the barriers to and facilitators of implementing teleconsultation in overnight nursing care.

### Data Collection

An interview guide was designed, tested, and validated by the research team. The guide, comprising 12 open-ended questions, aimed to identify the barriers to and facilitators of implementing teleconsultation. This guide provided a better understanding of the context and experiences surrounding the pilot project’s deployment. This study was guided by key factors influencing the implementation of health innovations as outlined in the framework proposed by Chaudoir et al [46]. The interview guide is included in Multimedia Appendix 1.

The framework developed by Chaudoir et al [46] was selected for several key reasons that ensure that it is an appropriate framework to assess the implementation of health innovations. In fact, this model is based on a systematic review of the literature. It also captures the complexity of implementing health innovations by considering various levels of influence, such as organizational structures, health care providers, patients, and the specific characteristics of the innovation.

This holistic approach enables a more comprehensive and nuanced assessment of the factors that can affect an innovation’s success. Furthermore, by incorporating levels of analysis that reflect practical realities in the field—such as the nursing staff and residents—the model provides health care professionals with an applicable and relevant framework. It helps identify the barriers and facilitators that are specific to each setting, thus facilitating the design of customized implementation strategies. Finally, the model is flexible and can be tailored to different health innovations and settings. Whether we are examining the implementation of a new technology or a care protocol, the model proposes analysis categories that can be adjusted according to the features of the innovation and environment.

The model’s components can be described on five levels: (1) the structural level, which includes factors related to the broader context in which the innovation is implemented, such as health care policies, funding structures, or available resources. These variables directly or indirectly influence an organization’s ability to adopt and integrate new practices. (2) the organizational level, which focuses on the specific characteristics of the organizations themselves, such as organizational culture, leadership, support systems, and communication dynamics. It examines how these internal elements facilitate or hinder the implementation of innovations. (3) the health care provider level; at this level, the focus is on the individuals responsible for implementation, such as physicians, nurses, or other health care professionals. The model assesses health care providers’ attitudes, knowledge, skills, and beliefs, all of which can influence how an innovation is adopted and applied. (4) the patient level, which analyzes patients’ perceptions, attitudes, and behaviors, as well as their level of innovation engagement and buy-in. Patients’ psychosocial factors, such as their understanding, beliefs, or preferences, are crucial to the successful implementation of health innovations. (5) the innovation level, which examines the specific characteristics of the innovation itself, such as complexity, compatibility with existing practices, cost, and flexibility. An innovation perceived as easy to use, relevant, and beneficial is more likely to be adopted.

This framework provides a solid foundation to analyze the perspectives of managers, nursing staff, and resident committee presidents, highlighting the key factors influencing the adoption and effectiveness of nursing teleconsultation in small CHSLDs.

### Participants

Participant recruitment was conducted using nonprobability sampling [47], through which participants were identified by pilot project managers in each region. This selection method is designed to maximize participants’ intrinsic motivation by giving them the opportunity to become involved on their own terms. By promoting this freedom of choice, this study aimed to attract participants who were especially motivated and engaged, thereby improving the quality of the collected data as well as enhancing the relevance and validity of the findings.

Participants were initially contacted through an email that included the interview guide and a detailed consent form. The consent form outlined the context of the study, the project’s objectives, the procedures and expected duration of participation, the anticipated benefits, and assurances regarding anonymity and confidentiality. A total of 38 semistructured individual interviews were conducted with managers (n=27, 71%), nursing staff (n=9, 24%), and resident committee presidents (n=2, 5%) between February 2023 and July 2023.

These semistructured interviews were carried out in French and were conducted via videoconference (Zoom; Zoom Video Communications, Inc). The principal investigator (VN) conducted the interviews. Participants were given the option to review the transcripts of each interview, but none of the participants chose to receive the transcripts. No additional recruitment process was necessary as information redundancy indicated data saturation [48].

### Data Analysis

On the basis of the framework by Chaudoir et al [46], we conducted a pattern analysis of interview transcripts to identify factors describing facilitators of and barriers to implementing overnight nursing teleconsultation in small CHSLDs. This approach emphasized hierarchical coding, enabling rigorous structuring of the textual data analysis while offering the flexibility required to meet the specific needs of the study.

The analysis prioritized participants’ responses, highlighting their descriptions of barriers and facilitators. To do this, we immersed ourselves in the data by reading and rereading transcripts while taking handwritten notes on emerging factors and codes. This iterative process carried out by a single coder (the principal investigator, VN) is a valid method in qualitative analysis of thematic data, enabling researchers to understand how participants gave meaning to their experiences. A handwritten thematic map was created to group data extracts into broad categories of barriers and facilitators, which fostered a thorough review process and helped generate initial codes. These codes were then applied during a second analysis phase using the NVivo software (version 14; QSR International). The initial codes were used to identify overlaps and search for emerging themes.

To ensure a comprehensive and structured analysis, the coder (VN) applied the 5 levels of the framework by Chaudoir et al [46]—structural, organizational, health care provider, patient, and innovation—as primary coding categories. Each identified barrier or facilitator was initially coded under one of these levels. The coder developed subthemes within each level, enabling a more nuanced examination of the data. This structured approach reinforced the consistency of our analysis, ensuring that all aspects of the implementation process were thoroughly explored while maintaining alignment with established theoretical constructs.

Finally, potential factors were examined and verified across the dataset by rereading the transcripts and checking themes against identified codes. This approach ensured the robustness and consistency of the findings.

### Ethical Considerations

Ethics approval was obtained from the Research Ethics Committee of the Outaouais Integrated Health and Social Services Centre before the beginning of the study (reference 2022-353_195) in Quebec, Canada. All participants gave their consent electronically before beginning the interviews. Participation was anonymous and voluntary. Study participants did not receive monetary compensation. All interviews were audio recorded with the participants’ permission and then transcribed in compliance with ethical and confidentiality standards. The deidentified recordings were transcribed verbatim by a third-party transcription service bound by a confidentiality agreement. The study’s findings will be disseminated through presentations at conferences and publications in peer-reviewed journals using anonymized data. The findings will also be shared through presentations to various MSSS stakeholders and the nursing community.

## Results

### Overview of Barriers

This study aimed to identify the barriers to and facilitators of implementing overnight nursing teleconsultation in 3 of Quebec’s rural CHSLDs with ≤50 beds. The results are presented in accordance with the 5 levels of the framework by Chaudoir et al [46]: structural, organizational, health care provider, patient, and innovation.

Table 1 presents a comprehensive overview of the framework detailing the barriers identified at each level and for each participant group.

**Table 1:** Level, factors, and number of participants who mentioned each factor.

Level and factor			Managers^a^ (n=27), n (%)		Nursing staff members^b^ (n=9), n (%)		Resident committee presidents^c^ (n=2), n (%)
**Structural**							
	Union opposition	23 (85)		—^d^		—	
	Network instability	11 (41)		4 (44)		2 (100)	
	Overburdened managers	16 (59)		—		—	
	Lack of support from project leaders	—		6 (67)		—	
**Organizational**							
	Lack of leadership from the site manager	—		4 (44)		—	
**Health care provider**							
	Resistance to change	12 (44)		6 (67)		—	
	Limited technology skills	12 (44)		7 (78)		—	
	Insecurity about using technology	12 (44)		4 (44)		—	
	Increased workload associated with the technology	10 (37)		8 (89)		2 (100)	
**Patient**							
	Concerns about quality of care	—		—		1 (50)	
**Innovation**							
	Low volume of teleconsultations	14 (52)		8 (89)		—	
	Complexity of the process compared to a phone call with a remote on-call nurse	16 (59)		—		—	
	Increased time to initiate care management	—		5 (56)		—	
	Insecurity about the quality of nursing assessments	—		5 (56)		—	
	Difficulty using the tablet	—		4 (44)		—	

^a^Individuals in positions of authority. They oversee operations, manage resources, and supervise personnel in health care settings. Their role is to ensure the smooth functioning of the facility.

^b^This refers to all individuals involved in providing direct care to patients or residents, including both nurses and nursing assistants.

^c^These individuals lead the residents’ committee. The committee represents residents in health care facilities such as long-term care homes. Its mandate is to protect residents’ rights. The committee ensures that residents are treated with dignity and that their rights and freedoms are respected. It also serves as a key spokesperson for residents. It brings residents’ concerns and needs to the attention of the institution’s governing bodies.

^d^Not applicable.

### Barriers: Structural-Level Factors

#### Union Opposition

Union opposition was mentioned by most managers (23/27, 85%) as a major factor before the launch of the pilot project. According to respondents’ testimonials, for several months, the union waged a disinformation campaign conveying alarming messages to health care professionals and the community.

The union disseminated messages stating that the “government was planning to replace nurses with tablets,” which it claimed would “diminish the quality of care and endanger the safety of residents” (M10). Another manager (M9) specified that “They ran a lot of ads saying nurses were being replaced by tablets*.*” Some managers reported that this campaign had a disruptive effect, creating “a shock wave and a wave of fear in the community” (M22), and the pilot project was perceived “as devaluing this client group, as if they were receiving second-class care” (M25).

The union’s intervention was not confined to the public sphere; it also manifested itself directly in the workplace. Managers reported that union representatives visited CHSLDs attempting to dissuade nurses from participating in the pilot project. One manager (M8) explained the following:

The union would come directly into the workplace, to frighten employees.

The union told nursing assistants that their participation was “super dangerous, because they would be going beyond their scope of practice and their professional order would turn against them,” added one manager (M9). Despite this initial pressure, none of the interviewed nursing staff members (9/9, 100%) reported any problems with their union after the beginning of the pilot project. Unlike the managers, nursing staff members did not perceive union opposition as a barrier.

#### Network Instability

Network instability was identified as a barrier by some managers (11/27, 41%) and nursing staff members (4/9, 44%), as well as by the resident committee presidents (2/2, 100%), especially in rural and remote areas.

Interviewees’ testimonials revealed that connectivity was not uniform across the facilities. One manager (M1) explained the following:

It didn’t necessarily work everywhere in the CHSLD.

In total, 44% (4/9) of nursing staff members added that there were sometimes 15- to 20-minute delays in logging in. This wasted time, although occasional, can have significant repercussions on the quality of care, as mentioned by one resident committee president (RC36):

From time to time, it won’t work there...you have to take that into account, because it would be a huge waste of time to start an intervention, then you lose the network, you have to restart another way, by telephone, etc.

#### Overburdened Managers

The extra workload associated with the pilot project was a barrier for 59% (16/27) of the managers, especially for project leaders, project coleaders, and site managers. They had to reconcile their usual tasks while ensuring rapid deployment of the project within the context of a nursing shortage. One manager (M4) explained the following:

We weren’t optimal in our monitoring, which created an obstacle, because, basically, we weren’t as present.

Moreover, daily monitoring of nursing staff practices increased the burden on site managers, who feared that implementation of the project would not be feasible without additional resources. One manager (M9) raised the following question:

Is the workload going to be realistic for managers who are already highly solicited?

#### Lack of Support From Project Leaders

Lack of support from project leaders was identified as a barrier by 67% (6/9) of nursing staff members. This limited support manifested as reduced availability as project leaders were often overwhelmed by their many responsibilities. One nursing staff member (NS34) indicated the following:

We’d have liked to have a little more time, but they’re kind of busy with everything.

This created a feeling of abandonment among nursing staff, with some expressing a lack of guidance. One staff member (NS29) reported that “The project leaders didn’t always have the answers to our questions.”

### Barriers: Organizational-Level Factors (Lack of Leadership From the Site Manager)

In one administrative region, the site manager’s lack of leadership was perceived as a barrier by some nursing staff members (4/9, 44%). This lack of leadership manifested itself as a lack of proximity between the site manager and the nursing staff, namely, infrequent travel to meet the team and gaps in communication. This distance hindered the exchange of information and the understanding of the pilot project’s issues, leading the site manager to become disinterested, which generated frustration and reduced nursing staff buy-in and motivation at the outset of the pilot project. However, as the project progressed, the situation improved, and they were able to overcome these challenges.

### Barriers: Health Care Provider–Level Factors

#### Resistance to Change

Resistance to change was identified as a barrier by 44% (12/27) of managers and 67% (6/9) of nursing staff members. This resistance took the form of a reluctance to participate in training and simulations, as well as a marked preference for using the telephone, which was perceived as quicker and more effective. For example, one nurse refused to use teleconsultation to assess a resident after a fall, preferring to travel to the CHSLD herself. One manager (M3) indicated the following:

The nursing staff found it cumbersome.

Indeed, nursing staff members reported that their colleagues preferred traditional methods such as on-call nursing or traveling to visit the resident in person. Even when teleconsultation would have been more appropriate, using the telephone remained the preferred method.

#### Limited Technology Skills

Limited technology skills represented a barrier, as identified by 67% (18/27) of the managers. One manager (M20) pointed out the following:

One of the barriers we encountered very, very quickly was that people were not familiar with the technology and then were not able to use it.

According to one manager (M23), this shortcoming can be explained by “a lack of simulation and comfort as well as by constraints such as nursing shortages, heavy workloads, and COVID-19 outbreaks.” One manager (M8) said the following:

In addition to learning new tools, our nurses had to continue providing care. At one point, they were saying, “It’s just not working”.... It was a real challenge to implement this on a daily basis.

According to 78% (7/9) of nursing staff members, this barrier is especially significant among older nurses, who are often less comfortable with new technology. One nursing staff member (NS33) described this generational challenge:

We weren’t all born with a keyboard or tablet in our hands...we have a few who are in their fifties. Not all of them were comfortable with it either.

One nursing staff member (NS29) added the following:

...although the younger generation showed an initial interest, this desire was curbed by older nurses’ reluctance to embrace the technology, creating a barrier to the successful integration of digital tools into professional practices.

Furthermore, the low volume of remote activities and insufficient monitoring limited the practice of teleconsultation, resulting in the loss of acquired skills. One nursing staff member (NS33) described it as follows:

We had practices, and I fell on the practice that was postponed.... It was never rescheduled. So, the first time, I’d never even practiced.

#### Insecurity About Using Technology

Insecurity about using technology was a concern due to the novelty of teleconsultation and was mentioned by 44% (12/27) of managers. In addition, 44% (4/9) of care staff members reported that nursing assistants in particular felt vulnerable when they were alone on-site, fearing that they would not know how to use teleconsultation properly or solve technical problems. As one manager (M4) explained, “It’s my nurse who takes charge” when a resident is not doing well. Another manager (M9) confirmed this feeling of insecurity:

This practice, in terms of the technology, well, it caused a little insecurity at first, because we didn’t know that much about it. 

Similarly, one nursing staff member (NS32) noted that “a fear of computer technology” discouraged some of the more experienced nurses from becoming involved in the pilot project.

#### Increased Workload Associated With the Technology

The implementation of teleconsultation led to an increased workload for remote nurses, a challenge that was highlighted by 37% (10/27) of managers, 89% (8/9) of nursing staff members, and 100% (2/2) of resident committee presidents. One nursing staff member (NS31) reported the following:

We were the ones who had to adapt the most. 

Before every night shift, nurses had to make sure that they had the necessary tools for teleconsultation, which often meant traveling to the CHSLD even on their days off. In addition to their usual tasks, they had to manage interdepartmental reports, fill in specific follow-up forms, and immediately document each teleconsultation. One resident committee president (RC36) explained the following:

We also had to foresee working time to connect, use the equipment, get everything working and then provide electronic notes afterward, transfer them, etc.

One manager (M23) pointed out the following:

...the people who are working remotely, the ones who are doing the teleconsultation, are the same people who are there in the evening, during the night...it creates a great deal of anxiety.

### Barriers: Patient-Level Factors (Concerns About Quality of Care)

In the context of teleconsultation, concerns about the quality of care were primarily raised by 50% (1/2) of the resident committee presidents. He feared that teleconsultation would undermine the personal nature of care, concerned that technology would compromise the human contact that is essential to in-person interactions. Initially opposed to the project, fearing the impersonality and disempowerment of nurses, he eventually recognized the benefits of teleconsultation as the pilot progressed. One of the presidents (RC36) expressed the following:

My initial opposition to the project was based on what I didn’t want: That it would become impersonal, that it would prevent human contact...that it would change the on-call nurse’s responsibility, relying on a screen, which is not the same as what you might experience during an in-person visit.

### Barriers: Innovation-Level Factors

#### Low Volume of Teleconsultations

According to 52% (14/27) of managers, the low volume of teleconsultations was a barrier and could be attributed to the small size of CHSLDs, but the low volume of activity also raised important questions, as one manager pointed out (M25):

Was the volume low because there wasn’t a need? Was the volume low because practices were already good on both sides, and [the person carrying out an intervention] acted preventively?

Another manager (M4) added the following:

Nursing assistants who said, “Oh no, look, it’s 4 o’clock. The nurse is coming in two hours, we’ll wait two hours.” Did this harm the resident? Well, indirectly, for someone who is in pain, yes it did. But there was no report of an incident or accident that could have or did cause harm to the resident’s health, safety, and well-being.

According to 89% (8/9) of nursing staff members, the low volume of teleconsultations hindered their ability to maintain their skills. One of them (NS26) said the following:

It had been a month since we’d had one...I forgot to fill in the smartsheet.

Moreover, a manager (M10) observed the following:

...when comparing data from the previous year to [the data related to] the implementation of teleconsultation, the number of telephone calls received is equal to the number of teleconsultations over the same period.

#### Complexity of the Teleconsultation Process Compared to On-Call Nursing

The complexity of the teleconsultation process compared to on-call nursing was perceived as a barrier by 59% (16/27) of managers. Unlike on-call nursing, when the nursing assistant can contact the on-call nurse directly, teleconsultation involves a series of more complex actions, such as waking up the on-call nurse to initiate the consultation and, sometimes, the need to call back the CHSLD. This complexity prolongs the time it takes to obtain a nursing assessment, as explained by one manager (M18):

It’s too slow...the time to turn on the laptop, to connect safely.

Another manager (M10) added that the speed of the on-call process influenced perceptions of teleconsultation:

It influenced the teleconsultation project.

In addition, the necessity of having the teleconsultation on-call travel case added another layer of difficulty, especially when staff forgot the equipment, as described by one manager (M12):

Ah OK, but now I don’t have the equipment, I have to go to the CHSLD to get the equipment.

#### Increased Time to Initiate Care Management

According to 56% (5/9) of nursing staff members, the use of teleconsultation led to an increase in the time taken to initiate the residents’ care management. Unlike previous practices, the nurse had to assess the resident over a digital platform before intervening, adding an extra step that delayed the response to immediate needs. This delay was exacerbated in remote areas, where unstable internet connections complicated access to teleconsultation, potentially leading to a deterioration of the resident’s condition. One nursing staff member (NS26) illustrated this problem by describing a situation in which the requirement to use a tablet for teleconsultation interfered with the prompt management of a resident’s pain:

A lady was experiencing pain.... I found the computer-based support detrimental to immediate care.... Meanwhile, the lady was in pain. You know, we are managing pain at the same time as we manage the tablet.

#### Insecurity About the Quality of Nursing Assessments

There were concerns about the quality of nursing assessments carried out via teleconsultation, including the fear that visual assessment cannot adequately replace a physical examination. In total, 56% (5/9) of nursing staff members shared these concerns. One of them (NS30) explained the following:

I had concerns about the physical assessment in the sense that shifting to a visual assessment instead of doing it in real life...that my assessment would not be complete. 

The absence of physical contact with the resident was perceived as a limitation as nonverbal communication plays an important part in a comprehensive assessment. Another nursing staff member (NS33) illustrated this difficulty:

Non-verbal and verbal [messages] contradict each other in residents...with the tablet, it wasn’t easy because I couldn’t look at my resident’s face and leg movement at the same time.

The use of technology such as the digital stethoscope also prompted reservations. One nurse expressed unease:

Listening over the phone, it’s not like performing the auscultation myself.

NS31

#### Difficulty Using the Tablet

When using a tablet, nursing staff members experienced physical constraints in terms of mobility and effectiveness. These constraints made it difficult to carry out teleconsultations, as one nurse described:

The hindrance was caused by the darn arm they set up to hold that tablet.... It would swing around. You know, to be honest, it wasn’t the best.

NS33

A total of 44% (4/9) of the nurses emphasized the need for a stand to hold the tablet, freeing up the nursing assistant’s hands.

### Overview of Facilitators

Table 2 presents a comprehensive overview of the framework detailing the facilitators identified at each level and for each participant group.

**Table 2:** Level, dimension, and number and percentage of participants who mentioned each factor.

Level and dimension		Managers^a^ (n=27), n (%)	Nursing staff members^b^ (n=9), n (%)	Resident committee presidents^c^ (n=2), n (%)
**Structural**				
	Care setting	21 (78)	9 (100)	—^d^
	Legitimization of the practice of overnight on-call nursing	18 (67)	—	—
	Culture of on-call nursing	—	—	2 (100)
	Implementation monitoring	21 (78)	—	—
**Organizational**				
	Buy-in from senior management and managers	27 (100)	—	2 (100)
	Support from project leaders	19 (70)	—	—
	Support from site managers	19 (70)	7 (78)	—
	Team involvement, motivation, and stability	15 (56)	—	—
	Collaboration between the nursing department and the Support Program for the Autonomy of Seniors	18 (67)	9 (100)	—
	Transfer of knowledge and experience	21 (78)	—	—
**Health care provider**				
	Nursing staff buy-in	13 (48)	—	—
	Nursing staff motivation	—	9 (100)	—
	Development of the nursing staff’s skills	13 (48)	—	—
	Ability to adapt and use technology	—	7 (78)	—
	Openness to change	—	—	2 (100)
**Patient**				
	Buy-in from residents, families, and resident committees	12 (44)	—	—
	Communication	—	6 (67)	—
**Innovation**				
	Relative benefits	27 (100)	9 (100)	2 (100)
	Development of nursing staff’s roles	19 (70)	—	—
	Improved professional practices	—	8 (89)	—

^a^Individuals in positions of authority. They oversee operations, manage resources, and supervise personnel in health care settings. Their role is to ensure the smooth functioning of the facility.

^b^This refers to all individuals involved in providing direct care to patients or residents, including both nurses and nursing assistants.

^c^These individuals lead the residents’ committee. The committee represents residents in health care facilities such as long-term care homes. Its mandate is to protect residents’ rights. The committee ensures that residents are treated with dignity and that their rights and freedoms are respected. It also serves as a key spokesperson for residents. It brings residents’ concerns and needs to the attention of the institution’s governing bodies.

^d^Not applicable.

### Facilitators: Structural-Level Factors

#### Care Setting

Testimonials from 78% (21/27) of the managers and all nursing staff members (9/9, 100%) highlighted the care setting’s decisive role in the success of the pilot project. Faced with a nursing staff shortage, this initiative was viewed as a promising solution to optimize practices and the management of available resources while maintaining the quality and safety of resident care. One manager (M1) explained the following:

Our objective is to ensure that every nursing staff member is in the right place, playing their role to the full and that we are using our resources wisely.

#### Legitimization of the Practice of Overnight On-Call Nursing

Legitimization of the practice of overnight on-call nursing factored positively in the project’s success. According to 67% (18/27) of managers, the fact that this practice was framed within a specific, temporary context reassured stakeholders such as the Order of Nursing Assistants of Quebec (Ordre des infirmières et infirmiers auxiliaires du Québec), the Quebec ombudsman, and user and resident committees. One manager (M1) explained the following:

This legitimization enabled nursing staff to feel they had greater authorization to use teleconsultation, mitigating fears related to professional compliance.

#### Culture of On-Call Nursing

The existing culture of on-call nursing was a significant facilitator. All resident committee presidents (2/2, 100%) stated that this culture, which was already well established and accepted in CHSLDs, facilitated the implementation of teleconsultation. Considered “an innovative and adaptive solution, on-call nursing was seen as essential to maintaining optimal quality of care,” as indicated by one of the presidents (RC36). He added the following:

...the pre-existing culture facilitated the transition to teleconsultation by normalizing the idea of a remote nurse and positioning it as a safe and effective approach.

#### Implementation Monitoring

Close monitoring by the nursing department (ND) and the Support Program for the Autonomy of Seniors (SAPA) within the health care system in Quebec was a key facilitator according to 78% (21/27) of managers. Monitoring took place at three levels: (1) strategic level—committees and regular meetings with the MSSS promoted fluid communication on project advancement; (2) operational level—project leaders organized regular meetings with site managers, enabling actions to be adjusted quickly and providing immediate feedback (as one manager [M16] reported, “monitoring by the Ministry...was highly beneficial”); and (3) day-to-day level—ongoing monitoring of teleconsultation practice was implemented, including a review of ministry forms, hospital transfers, and incident reports, as well as audits in CHSLDs to ensure that nursing staff had the support they needed.

### Facilitators: Organizational-Level Factors

#### Buy-In From Senior Management and Managers

Senior management buy-in was viewed as a facilitator by all managers (27/27, 100%), namely owing to the support of the chief executive officer of the Integrated Health and Social Services Centre of Abitibi-Témiscamingue and the Integrated University Health and Social Services Centre of Mauricie and Centre-du-Québec, as reported by one manager (M8):

He took it on, then he defended it.

When senior management prioritizes a project, it motivates other managers to engage, facilitating rollout and the resolution of challenges such as acquiring equipment—“We received our equipment very quickly because it was a priority,” according to one manager (M1). The resident committee presidents (2/2, 100%) also confirmed that this support was important to the project’s success. One of the resident committee presidents (RC35) explained the following:

When senior management is supportive of the project, it sends a strong signal to the members of the organization about the strategic importance of teleconsultation. This approval from leadership can positively influence the levels of acceptance and engagement within the team.

In addition, the managers’ buy-in was unanimously recognized as a facilitator by participating managers (27/27, 100%). Their engagement made it possible to effectively navigate MSSS requirements and ensure the buy-in of the nursing staff members who were consulted, underlining the importance of creating an environment that is conducive to the implementation of teleconsultation.

#### Support From Project Leaders

Support from project leaders was a determining factor according to 70% (19/27) of managers. Project leaders played a key role in motivating nursing staff by clarifying objectives, allaying concerns, and fostering champions within the teams, creating a conducive environment for the adoption of teleconsultation. As one manager (M5) explained, “Having a dedicated person to answer questions and solve problems” was essential.

#### Support From Site Managers

According to 70% (19/27) of managers, site managers also fostered the implementation of teleconsultation. One manager (M12) reported the following:

Their knowledge of the environment and their existing bonds of trust played a key role in the human management of change and in nursing staff mobilization.

The nursing staff (7/9, 78%) also appreciated this support, underlining the site managers’ guidance and availability. One nursing staff member (NS32) explained the following:

I felt supported throughout the project. If I had any questions, I knew where to turn. I had a lot of support from my manager.

#### Involvement, Motivation, and Stability of Nursing Staff

According to 56% (15/27) of managers, the involvement, motivation, and stability of nursing staff were key facilitators. Team cohesion facilitated flexibility and mutual support, as stated by one manager (M16):

Nursing staff demonstrated solidarity by swapping shifts during snowstorms to ensure staff availability. 

This solidarity enabled staff to respond effectively to residents’ needs and maintain reasonable response times.

#### Collaboration Between the ND and SAPA

According to 67% (18/27) of managers, collaboration between the ND and SAPA was a key facilitator. A clear division of roles enabled the ND to manage external relationships with the MSSS and other agencies, whereas the SAPA dealt directly with the implementation of teleconsultation, ensuring effective coordination. One manager (M16) reported the following:

Having a single point of entry was helpful.

This synergy promoted the cocreation of solutions to the project’s challenges, namely in terms of training and monitoring, reinforcing the effectiveness and success of the initiative.

The training plan, including coaching, simulations, and tool adaptations, was tailored to meet regional needs and reinforce the safety of nursing practices. One manager (M10) underlined the following:

Training was customized...to reinforce safety. 

Practical guides and equipment such as headsets supported the practice of teleconsultation, and simulations boosted the nursing staff’s confidence. Nursing staff members (9/9, 100%) unanimously appreciated the training, deeming it essential to the adoption of the technology and success of the pilot project.

#### Transfer of Knowledge and Experience

The transfer of knowledge and experience was a significant factor for most managers (21/27, 78%), facilitating collaboration and adaptation between regions and within participating CHSLDs.

Between regions, managers worked closely, sharing their experiences and adjusting approaches according to the specific needs of each region. Although support tools were not cocreated systematically, these exchanges enabled participants to adjust based on local context. Regarding collaboration and adaptation within CHSLDs, in one region, the level of sharing between 2 CHSLDs was particularly striking. Nursing staff from the first site shared their experiences with that of the second, fostering buy-in to the pilot project. For example, a manager’s guide created from lessons learned was passed on to the other site, facilitating the implementation of teleconsultation in similar settings. One manager (M16) described it as follows:

The manager took notes on everything she had implemented...and brought it back to the ND....So, it ranged from the criteria we had to meet, to making sure we met them, to the tasks we had to carry out...all in one guide.

### Facilitators: Health Care Provider–Level Factors

#### Nursing Staff Buy-In

For 48% (13/27) of managers, nursing staff buy-in was a facilitator. Nursing staff members were not only favorable to the idea, they were also motivated to actively participate in the pilot project, demonstrating a willingness to move forward with teleconsultation. One manager (M5) noted the following:

The nursing staff were very open and aligned with the project. They wanted to move forward with the change.

Despite initial stress, the nursing staff adapted quickly. As one manager (M24) pointed out, “Once the adaptation period was over...there was no more stress. It went well,” underlining their ability to overcome resistance and make a successful transition to teleconsultation.

#### Nursing Staff Motivation

All nursing staff members (9/9, 100%) considered that the motivation of health care staff was a facilitator. The main sources of motivation included commitment to the team, interest in technological tools, and the desire to help resolve the nursing shortage. Nursing staff members appreciated the creation of overnight nursing assistant positions with remote support, helping address the shortage and improve the quality of care. As one staff member put it, “We’re not as effective after 1 p.m” (NS28), highlighting the challenges of working long hours. Being motivated to use technological tools such as teleconsultation reflects a desire to explore innovative solutions to improve working conditions and better meet residents’ needs.

#### Development of the Nursing Staff’s Skills

Development of the nursing staff’s skills was a key factor, as highlighted by 48% (13/27) of managers. Training tailored to the staff’s needs fostered their preparedness and engagement, making them champions of the pilot project. Younger members showed greater mastery of computer and technological skills. In addition, the training improved nursing assistants’ level of autonomy and clinical judgment, contributing to the development of clinical leadership and team management skills. One manager (M7) described it as follows:

When she’s on her own, she also takes on a bit of a coordination role...something she didn’t usually do because it was part of the nurse’s role.

#### Ability to Adapt and Use Technology

For 78% (7/9) of nursing staff members, the ability to use technology was directly related to computer literacy and adaptability. Despite a low level of initial fluency, some managed to overcome their difficulties. One staff member (NS33) put it as follows:

I’m not very tech-savvy. I managed. It went well.

Thus, adaptability was a key factor, with nursing staff members demonstrating an ability to adjust to teleconsultation, including those who were not as comfortable with computers.

#### Openness to Change

Nursing staff’s openness to change was essential according to both resident committee presidents (2/2, 100%). This positive attitude was fostered by the fact that the project was recognized as an opportunity for exploration. One of the resident committee presidents (RC36) said the following:

Given that it was a pilot project, everyone agreed to give it a try.

RC35 added the following:

...the transfer of experience from nurses performing on-call nursing to teleconsultation is also a concrete example of this openness to change. The fact that the staff had prior experience in similar practices made it easier to adapt to new methods.

### Facilitators: Resident-Level Factors

#### Buy-In From Residents, Families, and Resident Committees

Buy-in from residents, families, and resident committees greatly facilitated the implementation of teleconsultation according to 44% (12/27) of managers. This support was reinforced by the creation of a relationship of trust through transparent communication and regular meetings with the site manager. One manager (M17) pointed out the following:

The site manager kept us informed on a regular basis, establishing a climate of trust.

#### Communication

Communication was a facilitator, especially in interactions with residents and their families. In total, 67% (6/9) of nursing staff members noted that proactive communication with residents and their families, namely, explaining the project, answering questions, and obtaining informed consent, facilitated resident buy-in. One nursing staff member (NS30) said the following:

Transparency, especially about concerns such as data leaks, fostered a positive reception to teleconsultation. 

Families generally welcomed the technology, recognizing the additional benefit to care delivery. Nursing staff members observed that there was no negative impact on residents, which could be attributed to the effective communication that reassured families about data confidentiality and security.

### Facilitators: Innovation-Level Factors

#### Relative Benefits

Analysis of the relative benefits of teleconsultation showed that this modality represented a significant added value for the dyad composed of the remote nurse and the CHSLD nursing assistant according to all managers (27/27, 100%), nursing staff members (9/9, 100%), and resident committee presidents (2/2, 100%). Benefits included, first, improved mutual vision. All nursing staff members (9/9, 100%) agreed that teleconsultation enabled direct observation of the resident and of the nursing assistant’s nonverbal cues, fostering a greater understanding of the situation than was possible through on-call nursing. One staff member (NS31) pointed out the following:

The way [the nursing assistants] report it to us over the phone and the way we see it through our own assessment, are two different things. Sometimes it’s minimized, and sometimes it’s exaggerated.

This visual component enhanced the remote nurse’s ability to carry out a more accurate and thorough assessment. One of the resident committee presidents (RC35) stated the following:

...the use of video in teleconsultation provides a clear advantage in terms of assessment quality compared to voice-only interactions. This ability to visualize the patient can lead to more informed decisions regarding necessary interventions.

The second benefit was faster assessment. Teleconsultation reduced the wait time for nursing assessments, enabling more effective interventions according to all nursing staff members (9/9, 100%). One staff member (NS32) noted the following:

...an intervention that might have taken an hour could take only 15 minutes with teleconsultation.

Moreover, the direct electronic transmission of nursing notes to the CHSLD promoted continuity of care. One of the resident committee presidents (RC36) noted the following:

...that despite the implementation of teleconsultation, it is not intended to completely replace in-person visits, but rather to complement care, thus offering flexibility and adaptability in the delivery of healthcare services.

The third benefit was improved quality of care management. The visual component of teleconsultation helped identify signs or symptoms that were not described verbally, leading to more informed decisions according to all nursing staff members (9/9, 100%). In addition, one manager (M25) reported that teleconsultation “provides visual support that the telephone does not, enabling [the nurse] to validate the on-site nursing assistant’s hypothesis or to support her in her contribution to the assessment*.*”

The fourth benefit was flexibility and safety. According to the resident committee presidents (2/2, 100%), teleconsultation did not replace in-person visits but was complementary to them, providing flexibility in the delivery of health care services. One of the presidents (RC36) mentioned the following:

Nurses are not prevented from physically traveling if necessary.

This innovation also contributed to resident safety according to the other president (RC35), noting that “residents were safe with this technological innovation, reinforcing the idea that teleconsultation does not entail any compromise in terms of patient safety.”

All managers (27/27, 100%) reported that the introduction of teleconsultation was perceived as a major step forward, enhancing the quality and safety of care delivery. According to them, teleconsultation provided greater safety by enabling nurses to exercise their clinical judgment under improved conditions. One manager (M4) stated the following:

Teleconsultation gives the manager a sense of security because they know that the healthcare professional is in a better position to exercise their clinical judgment.

#### Development of Nursing Staff’s Roles

The pilot project contributed significantly to the development of nursing staff’s roles according to 70% (19/27) of managers. Although overnight on-call nursing was already integrated into the organizational culture, the pilot project improved the organization and quality of care by strengthening nursing staff’s roles.

Key improvements included enhancement of the remote nurse and nursing assistant dyad as the nursing assistant’s practice was strengthened, including more effective nursing assessments and better care planning during the night shift, and expanded scope of practice for nursing assistants as changes to nursing care procedures enabled nursing assistants to fully exercise their skills. One manager (M1) pointed out the following:

Nursing assistants have activities...that we’ve allowed them to carry out.

Another manager (M16) added that the pilot project enabled them to “apply their entire scope of practice, enhancing the value and recognition of their role.”

#### Improved Professional Practices

The implementation of teleconsultation significantly improved professional nursing practices according to most nursing staff members (8/9, 89%). The main observed benefits were, first, the development of nursing assistants’ skills. Teleconsultation enabled nursing assistants to develop their scope of practice, acquiring new skills and playing a more active role in assessing and delivering care. Structuring communication when transmitting information also helped strengthen their communication skills. The second main observed benefit was proactive information updates. Teleconsultation facilitated the updating of therapeutic nursing plans and medication administration records, enhancing nursing assistants’ autonomy. One nursing staff member explained that teleconsultation eliminated the need to constantly contact the nurse for simple decisions such as administering medication, ensuring greater autonomy for nursing assistants. This staff member (NS29) underlined that “this project has really helped to make us more autonomous...we can manage almost everything on our own*.*”

## Discussion

### Principal Findings

#### Overview

The aim of this study was to gain a better understanding of the experiences of managers, nursing staff, and resident committee presidents involved in the pilot project to identify the factors that may promote the implementation of teleconsultation. Our multilevel analysis revealed the presence of facilitators and barriers.

#### Structural-Level Factors

Among the identified structural barriers, union opposition, which was reported by most managers (23/27, 85%), initially represented a significant obstacle, but it subsided once the project was in place. The union recognized that teleconsultation complied with professional standards while at the same time complementing the work done by nurses in CHSLDs without seeking to replace it. This helped dispel any initial fears. In addition, managers’ leadership played a decisive role by supporting the nursing staff, responding to their concerns, and facilitating a smooth and harmonious transition.

However, network instability in rural areas was a barrier for some managers (11/27, 41%) and nursing staff members (4/9, 44%). Connectivity issues coupled with the lack of overnight technical support led to delays and interruptions, compromising the effectiveness and reliability of teleconsultation. These difficulties highlighted the limitations of technological infrastructures in these environments and created situations in which interventions were delayed, compromising continuity of care. This problem is corroborated by studies that underline the importance of stable network connectivity to ensure the smooth operation of teleconsultation services. Indeed, network quality is a determining factor in ensuring the fluidity of exchanges and the effectiveness of remote consultations, as highlighted by several research studies [28,49-52]. In these settings, ensuring reliable network coverage and efficient technical support is imperative to avoid interruptions that could adversely affect the quality of care.

Overburdened managers, who had to juggle their usual responsibilities with the demands of the pilot project, led to suboptimal management according to 59% (16/27) of managers. This double workload, compounded by a nursing shortage, made it difficult to adequately monitor the project and led to management being less present and reactive. This finding is in line with observations found in the literature, which stress that work overload is a major challenge in project management, especially in settings with limited resources [53]. Thus, managers’ inability to respond optimally to project requirements due to the pressure of their day-to-day responsibilities contributed to the suboptimal implementation of the initiative, underlining the need for greater support for managers during the implementation of complex projects.

Lack of support from project leaders was a barrier for 67% (6/9) of nursing staff members. Insufficient training and monitoring limited the nursing staff’s ability to use teleconsultation effectively. This situation reflects the importance of organizational support, which studies have shown to be a key factor in the success of training and implementation programs [54]. Indeed, constant support and adequate coaching help build the staff’s skills and ensure the successful adoption of new technology, such as teleconsultation, in care settings.

However, several facilitators contributed to the project’s success. The context of the health care system, marked by a nursing shortage that was exacerbated by the COVID-19 pandemic, acted as a catalyst for the implementation of teleconsultation according to several managers (21/27, 78%) and all nursing staff members (9/9, 100%). Perceived as an effective solution to the shortage, teleconsultation enabled better management of human resources, which reduced excessively long shifts and improved the nursing staff’s working conditions. This approach is supported by studies demonstrating that technology can optimize the use of human resources during a shortage [55,56].

Some managers (18/27, 67%) highlighted that legitimization of the practice of overnight on-call nursing played a key role in nursing staff’s acceptance and adoption of teleconsultation. According to the literature, framing new clinical practices within a defined, transparent, and temporary framework is essential to reassuring stakeholders such as professional orders, user committees, and regulatory bodies [35,57,58]. Such a framework helps build an environment of trust, reducing reluctance to adopt innovative practices and ensuring compliance with professional standards.

The preexisting culture of on-call nursing facilitated the acceptance of the pilot project by making the transition to teleconsultation smoother according to the resident committee presidents (2/2, 100%). Familiarity with remote practices fostered acceptance of the new technology. This observation is supported by the literature, which indicates that preexisting practices and organizational cultures play a decisive role in the acceptance of health care innovations [35,59].

Finally, close monitoring at several levels was a key factor in the successful implementation of teleconsultation for a large proportion of managers (21/27, 78%). By integrating strategic, operational, and daily monitoring, the project benefited from rapid adjustments, ensuring compliance with project objectives and requirements. This systematic monitoring not only facilitated proactive management of challenges but also enhanced responsiveness to emerging issues, helping maintain coherent and fluid processes. According to the literature, structured and sustained monitoring is essential to optimizing the implementation of new practices, especially in a context of technological transformation, helping overcome barriers and ensure project sustainability [60,61].

#### Organizational-Level Factors

According to the nursing staff in 1 of the 2 administrative regions (4/9, 44%), one of the barriers at the organizational level was the site managers’ lack of leadership. This lack of leadership, characterized by minimal presence and limited communication with nursing staff, was viewed as a barrier to the implementation of teleconsultation. The perceived distance between the site managers and care teams created a climate of frustration and disengagement. This situation is corroborated by the literature, which underlines that weak leadership can hinder the implementation of change initiatives by generating feelings of frustration and disengagement among health care teams [62-64].

As part of the implementation of teleconsultation, several organizational factors facilitated its rollout, highlighting the importance of a coordinated approach and sustained engagement at all levels of the organization. Buy-in and active support from senior management and managers were crucial to the project’s success according to all managers (27/27, 100%) and resident committee presidents (2/2, 100%). The literature supports this observation, stating that the engagement of organizational leaders is a key factor in the success of change initiatives in health care facilities [65,66]. This engagement helped mobilize the necessary resources and promote a culture conducive to innovation.

The role of project leaders and site managers was considered essential by 70% (19/27) of managers. Their availability and expertise not only enabled the effective resolution of operational issues but also played a key role in maintaining the nursing staff’s level of motivation.

Specifically, nursing staff members (7/9, 78%) stated that regular meetings and personalized support helped clarify objectives, allayed concerns, and ensured constant monitoring, which helped overcome challenges and optimize implementation processes. These results are in line with the literature, which emphasizes the importance of management practices and managers’ commitment to the success of digital transformation initiatives [67,68].

According to some managers (15/27, 56%), the involvement, motivation, and stability of nursing staff members were also facilitators. The staff’s ability to adapt and maintain a high level of service despite challenges was facilitated by increased motivation and cohesiveness, which is supported by the work by Nabelsi et al [35]. Their research shows that staff motivation and stability are essential elements to ensuring efficient processes in health care. The solidarity and cooperation observed within the teams helped maintain high levels of service even under difficult conditions.

Another facilitator was the collaboration between the ND and the SAPA according to all nursing staff members (9/9, 100%) and several managers (18/27, 67%). This collaboration clarified the division of roles, avoided duplication, and ensured smooth project management. A clear division of responsibilities and coordination between departments are essential [35]. This model of cross-directorate collaboration helped maximize efficiency and avoid overlaps that might slow down the implementation process.

Finally, most managers (21/27, 78%) said that the transfer of knowledge and experience between regions and within CHSLDs played a significant role in localizing the project. This approach, which centered on collaboration and the sharing of best practices, enhanced the effectiveness of the pilot project [35]. The study by Nabelsi et al [35] shows that knowledge sharing between teams and sites not only fosters the adoption of technology, it also enables better management of the challenges encountered in the field by adapting to local needs and constraints.

#### Health Care Provider–Level Factors

Resistance to change was a barrier to the implementation of teleconsultation, especially for certain nursing staff members (6/9, 67%). Their marked preference for the telephone, perceived as faster and more effective, highlighted their resistance to the adoption of teleconsultation. The literature on organizational change in health care indicates that this resistance may be fueled by the perception of being overloaded and by deeply ingrained habits, making it difficult to accept new technology [28,69,70].

According to nursing staff members (7/9, 78%), a lack of skill in using new technology was also a limiting factor, especially among older nursing staff members, who were not as comfortable with technological tools. This shortcoming is a recognized factor in the failure to implement technology in health care [44,71,72]. Work overload, exacerbated by crises such as the COVID-19 pandemic, added a further dimension to this challenge, creating a cognitive overload that complicated the effective integration of new technology.

Insecurity about using technology was another barrier for a small proportion of managers (12/27, 44%) and nursing staff members (4/9, 44%). Nursing assistants in particular felt vulnerable due to their lack of familiarity with teleconsultation and fear of dealing with technical problems. The literature on technology acceptance underlines that insecurity and fear of the unknown can reduce user motivation and performance [73,74].

The increased workload associated with technology was also a barrier. The implementation of teleconsultation led to increased management of reports, specific forms, and detailed documentation, an issue recognized by most nursing staff members (8/9, 89%) as well as all resident committee presidents (2/2, 100%). Studies show that increased workloads can cause stress and reduce job satisfaction, negatively influencing the implementation of new practices [75,76].

Among the facilitators, nursing staff buy-in was important, although it was mentioned by a lower proportion of managers (13/27, 48%). The nursing staff’s willingness to participate in the pilot project and their positive attitude toward teleconsultation were key to its success. The literature on organizational change in health care suggests that stakeholder buy-in is important to the success of change initiatives [77,78].

Nursing staff members’ motivation also played a significant role in the pilot project’s success, a factor unanimously recognized by all staff members (9/9, 100%). Commitment to the team, interest in technological tools, and the desire to help solve the nursing shortage were all motivating factors. Research shows that motivation is a facilitator of the acceptance and successful use of health technology [79,80].

Development of the nursing staff’s skills was a facilitator, although it was mentioned by a lower proportion of managers (13/27, 48%). Adequate training strengthened nurses’ readiness and commitment, enabling them to become project champions. The literature indicates that skill development is key to the successful adoption of new technology [66,81,82]. Customized training and support tools such as practical guides and simulations helped build the nursing staff’s skills and confidence [35].

The ability to use the technology was also crucial to the success of the pilot project, as underlined by nursing staff members (7/9, 78%). Rapid adaptation to new technology is indeed a facilitator, as confirmed in research on technology acceptance [74,80].

Finally, openness to change facilitated the implementation of the project, a factor that was unanimously recognized by resident committee presidents (2/2, 100%). This openness to change led to smoother adoption of teleconsultation, which is supported by studies demonstrating that acceptance of change is essential to the success of transformation initiatives [83].

#### Resident-Level Factors

The only barrier was fear concerning the quality of care provided via teleconsultation, which was mentioned by 50% (1/2) of the resident committee presidents. One of the presidents expressed concerns about the reduction in human contact, which could lead to perceived depersonalization of care. However, these concerns were dispelled as the pilot progressed, and he eventually recognized the benefits of teleconsultation. This fear that technology would disempower nurses and create a sense of impersonality represented a barrier to the acceptance of teleconsultation. Studies indicate that concerns about quality of care and depersonalization can negatively influence acceptance of telehealth technology by residents and their families [50,72,84,85].

According to some managers (12/27, 44%), buy-in from residents, families, and resident committees was a facilitator of the implementation of teleconsultation. The managers observed that transparent communication and regular meetings with these stakeholders helped establish a climate of trust. This approach is in line with the literature, which stresses the importance of trust and effective communication when fostering acceptance of health technology by patients and their families [86-88].

For most nursing staff members (6/9, 67%), effective communication itself was a facilitator. Research shows that managing expectations and clarifying the benefits of new technology are important to their acceptance by residents and their families. The ability to clearly explain the benefits of teleconsultation and address residents’ concerns contributed greatly to their buy-in [35].

#### Innovation-Level Factors

The low volume of teleconsultations was recognized as a barrier by more than half (14/27, 52%) of the managers. Several possible explanations were put forward. Some managers suggested that this low volume may reflect a lack of real need, the preexisting effectiveness of preventive practices, or some nursing staff members’ reluctance to use teleconsultation. However, when comparing data from the previous year with the data related to implementation of teleconsultation, findings reveal that the number of telephone calls received was equal to the number of teleconsultations over the same period. In addition, the low volume of consultations impacted the nursing staff’s ability to maintain their skills. Most nursing staff members (8/9, 89%) reported a decrease in their level of comfort with the technological tools due to sporadic use.

The complexity of the teleconsultation process was also a barrier for more than half (16/27, 59%) of the managers. Compared to traditional on-call nursing, teleconsultation involves more complex technological processes. Connolly et al [89] underline that this complexity can reduce the effectiveness of interventions and increase staff frustration, hindering the adoption and effectiveness of technology [51,90].

Another barrier was the increased time to initiate care management according to over half (5/9, 56%) of nursing staff members. Using digital platforms for assessments can increase response times, a problem that is exacerbated by technological limitations and connectivity issues. The research by Pilosof et al [91] shows that these delays can adversely impact the quality of care by affecting the responsiveness of interventions.

Concerns about the quality of assessments conducted via teleconsultation were noteworthy. Just over half (5/9, 56%) of nursing staff members expressed concern about the ability of visual assessment to effectively replace a physical assessment, highlighting the potential risk of compromising quality of care. This fear is corroborated by studies revealing that telemedicine can sometimes alter the quality of clinical assessments if not properly integrated into care practices [85-87,92-95].

Finally, the difficulty of using a tablet for teleconsultations represented a barrier for a small proportion of nursing staff members (4/9, 44%). Srinivasan et al [85] highlight that ergonomic issues and difficulties in handling technological equipment can reduce the effectiveness of interventions and user satisfaction, complicating the integration of teleconsultation [86,87,89].

Despite these challenges, several innovation-level factors facilitated the implementation of teleconsultation. The relative benefits of this technology were viewed positively by all managers (27/27, 100%), nursing staff members (9/9, 100%), and resident committee presidents (2/2, 100%). They appreciated the improved mutual vision and faster assessment. Teleconsultation solutions offer significant benefits in terms of visual communication and speed of intervention [35]. This perception of the benefits fostered acceptability and support for the project.

The development of nursing staff’s roles was another facilitator according to several managers (19/27, 70%). The project strengthened professional practices and broadened the nursing assistants’ skills. The work by Nabelsi et al [35] indicates that teleconsultation technology can support the expansion of professional roles and improve quality of care.

Moreover, the implementation of teleconsultation led to improved professional practices for most nursing staff members (8/9, 89%). Research has shown that technology integration improves professional skills and care management, highlighting the potential of innovations to positively transform practices in care settings [35,91,96].

### Limitations and Future Research

This study has a number of limitations that must be taken into account when interpreting the results. First, this study was conducted in only 2 Quebec regions, including a total of 3 CHSLDs. This limited scope may restrict the generalizability of the results to other geographical settings or types of facilities. In addition, the small number of sites included in the study may not enable researchers to capture the diversity of practices and challenges encountered in other regions or in facilities of different sizes. Second, this study focused exclusively on smaller facilities with ≤50 beds. While this is in line with the study’s objective of targeting small CHSLDs, the results may not be directly applicable to larger facilities, which may have different organizational structures and needs. Finally, this study’s evaluation period was short, making it impossible to observe the long-term impact of nursing teleconsultation, especially in terms of the continuous improvement of nursing practices, the sustainability of interventions, and the changes in stakeholder perceptions.

Furthermore, this study used a nonprobabilistic sampling method, which may have resulted in the inclusion of participants who were more inclined to view teleconsultation favorably or who had a particular interest in the topic. To mitigate this potential selection bias, the team researcher actively sought a diversity of perspectives during data collection, encouraging participants to share both supportive and critical viewpoints regarding the implementation of teleconsultation. Moreover, a rigorous qualitative analysis was conducted, with particular attention given to dissenting opinions and negative experiences, ensuring a comprehensive representation of the facilitators and barriers encountered. Despite these efforts, the inherent limitations of qualitative research, including the subjectivity of self-reported experiences, necessitate further investigation through complementary methodologies. A quantitative study conducted through a survey would strengthen the robustness of our findings and allow for a more generalizable assessment of the impact of teleconsultation

To broaden our understanding of the implementation of nursing teleconsultation in long-term care, it would be relevant to conduct studies in a larger number of CHSLDs, including facilities of various sizes located in different regions, to assess the transferability of this study’s findings and their effectiveness on a larger scale. Longitudinal research would also be needed to assess the long-term effects of nursing teleconsultation on quality of care, resident satisfaction, and human resource management in CHSLDs, as well as to identify any adjustments needed to ensure the sustainability of these practices.

It would also be useful to study the impact of teleconsultation on nurses’ well-being and workload, namely by examining how this technology can be optimized to effectively support their role without increasing their stress level or mental load. Finally, economic studies could help quantify the costs associated with implementing nursing teleconsultation and compare them with potential savings in terms of decreased hospitalizations, adverse events, and improved quality of care.

### Conclusions

This study provides the first in-depth analysis of barriers and facilitators related to the implementation of overnight nursing teleconsultation in small long-term care facilities. The findings provide a better understanding of these barriers, which can be used to develop strategies to overcome them during implementation. These findings are also particularly relevant to decision makers who are responsible for designing health initiatives as their choices influence the implementation and scaling-up process.

Broadly, the results provide a comprehensive overview of the factors influencing the successful implementation of teleconsultation in long-term care. This can help identify key factors to consider when scaling up teleconsultation in CHSLDs. The framework developed by Chaudoir et al [46] highlights the concept of adaptability, emphasizing the importance of adjusting the deployment of an innovation to suit the specific context. When scaling up teleconsultation, it is important to consider the specific characteristics of each CHSLD and region and tailor the implementation of teleconsultation accordingly.

While resistance to change is often considered a major barrier to implementing new health care technologies, our findings challenge this assumption. In the rural CHSLDs studied, the preexisting on-call nursing culture not only facilitated the adoption of teleconsultation but also eased its integration into clinical practice. This contrasts with previous research suggesting that health care professionals may resist new technology due to concerns about workflow disruptions or unfamiliarity with remote care models. In this context, previous experience with remote support likely mitigated these challenges, highlighting the importance of accounting for contextual factors when implementing teleconsultation. Furthermore, the identification of a low volume of teleconsultations as a barrier contradicts the common assumption that a phased rollout is always beneficial. Instead, our results suggest that achieving a critical mass of teleconsultations is essential to maintaining staff engagement and competencies. These findings provide new insights into teleconsultation implementation by demonstrating how preexisting practices and use patterns can significantly influence the adoption and sustainability of technological innovations in long-term care settings.

Efforts to implement overnight nursing teleconsultation in long-term care are more likely to succeed if they are based on an understanding of the forces driving the dissemination and scale-up of teleconsultation. Therefore, further research is needed to develop and strengthen the conceptual and applied foundations of the dissemination and scale-up of health care innovations, especially in the context of Quebec’s emerging learning health care systems.

## Appendix

Multimedia Appendix 1 Interview guide.

## Abbreviations

CHSLD: centre d’hébergement et de soins de longue durée
MSSS: Ministère de la Santé et des Services sociaux
ND: nursing department
SAPA: Support Program for the Autonomy of Seniors
