# Supplementary material for: Barriers to and Facilitators of Implementing Overnight Nursing Teleconsultation in Small, Rural Long-Term Care Facilities: Qualitative Interview Study
Source: JMIR Aging. 2025 May 7;8:e71950. doi: 10.2196/71950 (PMC12096027; doi:10.2196/71950)
Supplement: Multimedia Appendix 1 [file aging_v8i1e71950_app1.docx]

**Multimedia Appendix 1 – Interview guide**

1. As a participant from CIUSSS MCQ/CISSSCA, what is your role in the deployment of teleconsultation in small residential and long-term care centres (CHSLD)?
2. General question: Since you have been involved in the implementation, how is the deployment of innovations (organizational/technological) in CHSLD going?
3. According to your perception, where do you stand with the implementation of innovations (organizational/technological) in CHSLD?
4. What changes in practice have you observed as part of the deployment in CHSLD?
5. What are your expectations and concerns regarding the implementation of these innovations (organizational/technological)? Now, we will discuss the various favorable elements and obstacles to the implementation of innovations (organizational/technological) in CHSLD.
6. In your opinion, what factors facilitate the implementation of innovations at your site...?
7. In your opinion, what factors hinder the implementation of innovations at your site...?
8. What challenges do you face in your role (nurse or nursing assistant) in the deployment of teleconsultation in CHSLD?
9. Are there any elements that have not been addressed that you would like to discuss?
